# Multiplexed Detection of Membranous Nephropathy Antigens by Multi-Reaction Monitoring Mass Spectrometry

**DOI:** 10.1016/j.ekir.2026.106561

**Published:** 2026-04-22

**Authors:** Aaron J. Storey, Tiffany N. Caza, Samar I. Hassen, Owen W. Stephens, Lindsey Vanhouten, Rick Edmondson, Daniel Kenan, Christopher P. Larsen

**Affiliations:** 1 Arkana Laboratories, 10810 Executive Center Drive, Little Rock, Arkansas, USA; 2 Department of Nephrology, University of Arkansas for Medical Sciences, Little Rock, Arkansas, USA

**Keywords:** antigen, autoantigen, kidney biopsy, kidney pathology, mass spectrometry, membranous nephropathy

## Abstract

**Background:**

Membranous nephropathy (MN) is a leading cause of nephrotic syndrome, driven by an immune response against a target autoantigen. To date, over 30 target antigens have been described in MN, creating the need for multiplex approaches. We developed a multiple reaction monitoring (MRM)-based assay for the clinical mass spectrometry (MS) laboratory for determination of an antigen type in nephropathology practice. Here, we provide detailed methods and validation data to ensure adaptability among different instruments and laboratories.

**Methods:**

Protein A/G immunoprecipitation was used to extract immune complexes from frozen kidney biopsy tissue. Proteins were eluted from magnetic beads, reduced, alkylated, and trypsin digested using single-pot, solid-phase-enhanced sample preparation workflow. Tryptic peptide quantities were measured by MS with internal standard (IS) peptides. Peak area ratios were used to call an antigen type. Immunostaining was used as an independent method to confirm target antigen types. A calling algorithm was empirically developed that maximizes sensitivity and specificity of the workflow for identifying membranous antigens in kidney biopsies.

**Results:**

Our immunoprecipitation-to-MS workflow demonstrated reliable quantitative detection of a total of 19 MN antigens. The MS-enabled workflow had a diagnostic accuracy of 97.2% with a sensitivity of 97.2% and 100% specificity from a validation cohort of 113 MN cases with antigen type confirmed by immunostaining.

**Conclusion:**

MRM-MS following protein A/G immunoprecipitation of MN biopsy tissue can be effectively used for multiplex antigen typing in clinical nephropathology practice.

## Graphical abstract

**Figure:**
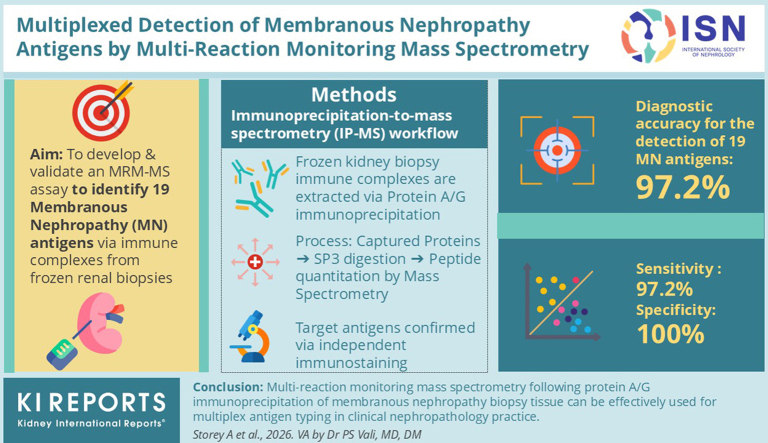

MN is an autoimmune kidney disease characterized by glomerular deposition of immune complexes containing specific protein antigens. In the majority of cases, the autoantigen is identified through immunostaining of kidney biopsies, with the exception of phospholipase A2 receptor (PLA2R)-positive MN which can be identified either on biopsy or serologic testing.^1^ More than 30 antigens have been identified to date.^2^, ^3^, ^4^

Many MN antigens have known disease associations, including neural epidermal growth factor-like 1 (NELL1) and thrombospondin type 1 domain containing 7A (THSD7A) with malignancy,^5^^,^^6^ NELL1 in exposure to certain drugs^7^^,^^8^ and mercury,^9^, ^10^, ^11^ proprotein convertase subtilisin/kexin type 6 (PCSK6) with nonsteroidal anti-inflammatory drugs use,^12^ neuron-derived neurotropic factor (NDNF) with syphilis,^13^ exostosin 1/2 (EXT1/2) and neural cell adhesion molecule-1 (NCAM1) with systemic lupus erythematosus,^14^, ^15^, ^16^ among others. Knowledge of the autoantigen can guide clinical testing to find an underlying etiology, and in some cases, identify a reversible cause. Another advantage of identifying an antigen is to monitor disease activity through serologic testing. These are currently available for MN positive for PLA2R or THSD7A. Currently, serologic tests are not available for the majority of antigens, however, many have been shown to have evidence of circulating antibodies in proof-of-concept studies, opening the door for future noninvasive monitoring.

The current standard of care of MN antigen typing consists of immunostaining for various antigen types on biopsies. This has become impractical in practice, particularly for PLA2R-negative cases, due to the large number of antigens. Therefore, limited typing is typically performed and many MN cases remain of unknown antigen type. Therefore, multiplexed approaches are urgently needed for MN antigen typing both to facilitate workflows in the clinical laboratory, as well as to provide more patients with a precise diagnosis.

An MRM-MS platform provides selective and quantitative detection of target proteins in biological samples and can serve as an excellent method for multiplexed detection of MN antigens. MRM assays have the advantage of being standardized and reliably reproduced among laboratories and across instruments. In this article, we provide detailed methods for an MRM-MS assay for typing of MN cases in the clinical laboratory and report its performance characteristics.

## Methods

### Case Selection

Frozen kidney biopsy tissue remnants were obtained from the biorepository at Arkana Laboratories following institutional review board approval. Cases were identified from a biopsy database from 2022 to 2025 that were diagnosed with MN and membranous lupus nephritis (MLN). Biopsies of known MN antigen types were selected where immunostaining performed at the time of diagnosis in our routine clinical workflow, including cases positive for PLA2R, THSD7A, NELL1, EXT1/2, NDNF, NCAM1, and myeloperoxidase.

MN or MLN cases of additional rare antigen types were identified through screening cohorts of PLA2R-negative cases by MS, followed by immunostaining verification of target(s). Cases within validation cohorts met minimum adequacy criteria of 8 glomeruli within the frozen biopsy tissue.

### Target Protein Antigen Analytes

A total of 19 proteins were included in our panel. Known antigens included PLA2R, THSD7A, EXT1/2, NELL1, NCAM1, myeloperoxidase , NDNF, protocadherin 7 (PCDH7), heat temperature-related protein 1 (HTRA1), semaphorin 3B, transforming growth factor beta receptor 3, protocadherin FAT1, proprotein convertase subtilisin/kexin type 6 (PCSK6), contactin 1 (CNTN1), and netrin G1. Putative antigens included cystine-rich motor neuron 1 protein (CRIM1), macrophage-stimulating protein, seizure-related 6 homolog 2, and vasorin.

### Selection and Verification of Target Peptides

Recombinant proteins or human embryonic kidney-293T cell over-expression protein lysates were obtained for all antigen proteins (Supplemental Methods). Two to 4 unique peptides for each protein antigen were identified from the transfection lysate and/or recombinant protein digests. Each IS was analytically validated by the Clinical Proteomic Tumor Analysis Consortium Guidelines,^17^^,^^18^ with details of performance characteristics (limits of detection, inter- and intra-assay variability, and matrix effects) included in Supplementary Tables S1 and S2.

### Multiplex Panel Construction for Internal Standard Peptides

A solution was prepared containing absolute quantitation grade isotopically heavy IS peptides for each target analyte. An equivalent solution was prepared containing isotopically light peptides for each analyte. The mixture of heavy peptides is included in every unknown sample for estimation of endogenous peptide molecules liberated from the tryptic digest. IS peptides were injected at 5 fmol/MS run.

### Multiple Reaction Monitoring

Peptide samples were analyzed by MRM on a TSQ Altis (Thermo Fisher Scientific , Waltham, MA) interfaced with a Waters M-Class (Waters Corp, Milford, MA) liquid chromatography. Peptides were direct loaded on a heated (55 °C) EasySpray column (150 mm x 75 μm, 3 μm, ES900 [Thermo Fisher Scientific , Waltham, MA]) at 1.2 μl/min for 6 minutes and eluted at 400 nl/min over a 27.5 minute separation gradient (detailed in Supplementary Table S3). Peptide detection limits were established during analytical verification experiments and use ion ratios of confirming ions, ion coelution values between target peptide and IS, and quantitative limits of detection established by external calibration curves. For figure construction, peak area ratios and isotope dot product values were exported from Skyline and processed using R (Posit PBC, Boston, MA).

### Immunoprecipitation and Peptide Preparation

Frozen kidney biopsy tissue remnants were washed in phosphate buffered saline, followed by bead-beating in 400 μl Pierce IP lysis buffer (Thermo Scientific #87787) containing protease and phosphatase inhibitors (Halt protease/phosphatase inhibitor at 1:100 dilution, Thermo Scientific #87786) to produce protein extracts. Protein extracts were immunoprecipitated with protein A/G beads (Pierce #88803) in the presence of 50 ng stable isotope-labeled universal monoclonal antibody (SILuMab K1;Sigma-Aldrich #MSQC6), and 50 ng SILuMab K4 (Sigma-Aldrich #MSQC7). Samples were then washed and transferred into 100 μl bead elution buffer (Tris 100 mM pH 8.0, 0.2% SDS, 5 mM dithiothreitol), followed by alkylation of cysteine residues with the addition of 15 mM iodoacetamide. Samples were spiked with 5 fmol IS peptides for each monitored peptide (per injection). Peptide samples were desalted using KT-Coll-μ96W-PurePep-Broad.H.1 desalting plates (Affinisep, France), dried by centrifugation in a SpeedVac, and reconstituted in 50 μL Buffer A (0.1% formic acid, 0.5% ACN). Reconstituted samples were analyzed in 5 μL injections.

### Immunostaining for Confirmation of Antigen Type

Immunohistochemistry or paraffin immunofluorescence was performed for confirmation of antigen type in all cases. Antibodies and staining conditions for all antigen types are included in the Supplementary Methods.

## Results

### Target Antigens Evaluated in the MRM-MS Assay

A total of 19 protein antigens were included in our panel. Up to 4 peptides were obtained and validated for each of the protein antigens, including PLA2R, THSD7A, EXT1/2, NELL1, NCAM1, myeloperoxidase , NDNF, PCDH7, HTRA1, semaphorin 3B, transforming growth factor beta receptor 3, protocadherin FAT1, PCSK6, CNTN1, netrin G1, CRIM1, macrophage-stimulating protein, seizure-related 6 homolog 2, and vasorin. Peptides were evaluated for quantitative assay performance through measurement of peak area ratios of external validation curves.

### Procedure Overview

**Figure 1:**
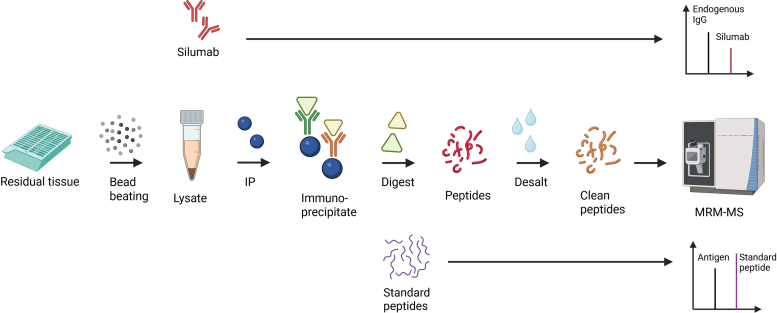
Overview of the MRM-MS MN assay procedure. Protein lysates are prepared from residual frozen kidney biopsy tissue by mechanical disruption through bead-beating. Lysates are immunoprecipitated with protein A/G beads to recover immune complexes, with an immunoglobulin standard added during immunoprecipitation to serve as a surrogate for IgG recovery. Immunoprecipitated proteins are digested off beads and standard peptides are added at the time of the digest for downstream antigen quantitation. Peptides are desalted and cleaned, followed by MRM-MS of a 19-antigen panel for quantification of target antigen(s). Further detail is provided in the Methods. MN, membranous nephropathy; MRM-MS, Multiple reaction monitoring - mass spectrometry.

To determine the antigen type of MN cases, immune complexes were extracted through protein A/G immunoprecipitation of protein lysates derived from remnant frozen biopsy tissue. Samples used were 1 to 3 years postbiopsy, although we have successfully identified antigen type by this method in cases frozen for up to 10 years. For assessment of pulldown efficiency and measure the quantity of recovered immune complexes, an isotopically labelled immunoglobulin (SILuMab) was spiked into the lysate. Tryptic digests were made from protein A/G immunoprecipitates to prepare endogenous peptides, which were spiked with 5 fmol IS peptides. IgG and IS recovery were measured for sample adequacy. Sample adequacy was overall dependent on immune complex recovery, rather than the total protein concentration included in the input lysate before immunoprecipitation (with typical yields of 150–600 μg protein in input lysates). An overview of the workflow is shown in Figure 1.

### Establishment of a Calling Algorithm

In order to implement the workflow as a clinical test, a defined procedure is necessary to call a definitive antigen type from the acquired MS data. Thus, a calling algorithm was empirically developed to establish criteria for antigen calls and to evaluate assay performance. The algorithm was produced based on the number of peptides detected, amount of IgG captured, and IS recovery. The average peptide quantities (in fmol) were assessed in immunostaining confirmed cases to establish cutoffs for positive calls.

Frozen residual kidney biopsy samples MN samples of known antigen type from immunostaining were processed by the pulldown-MRM workflow (Figure 1) and the acquired MS data was analyzed in Tracefinder (Thermo Fisher Scientific, Waltham, MA). For each sample, the number and quantity of detected antigen peptides (in fmol) are obtained, along with the quantity of endogenous IgG (in ng) and the intensity of a monitored SILuMab peptide generated from the tryptic digest (in ion counts). SILuMab standards enable an assessment of immunoprecipitation efficiency and immunoglobulin capture. IgG capture was assessed through measurement of a peptide within SILuMab unique to other IgG molecules.

**Figure 2:**
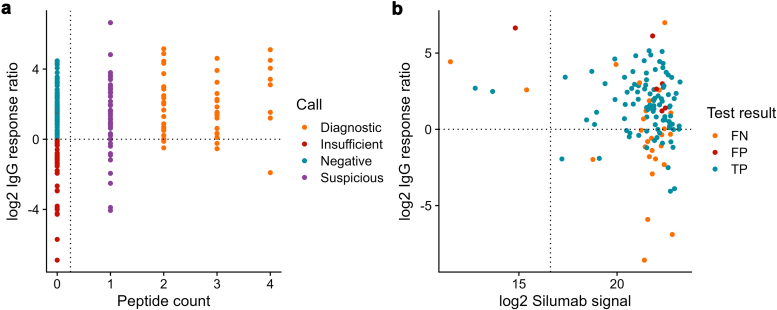
Determination of IgG quantity and internal standard cutoffs for sample sufficiency. (a) Comparison of PLA2R/IgG response ratio and peptide count in PLA2R+ MN samples. Samples with > 1 peptide provided diagnostic results. (b) Developing quality cutoffs for a calling algorithm. Plot showing initial calling algorithm performance on pre-validation samples with varying levels of IgG and internal standard (IS) recovery. Points are denoted as true positive (TP), false positive (FP), or false negative (FN) based on concordance with immunostaining. Domains were defined based on IgG and IS cutoffs, and calling algorithm accuracy was evaluated for data points within these domains. MN, membranous nephropathy; PLAR2, phospholipase A2 receptor.

**Figure 3:**
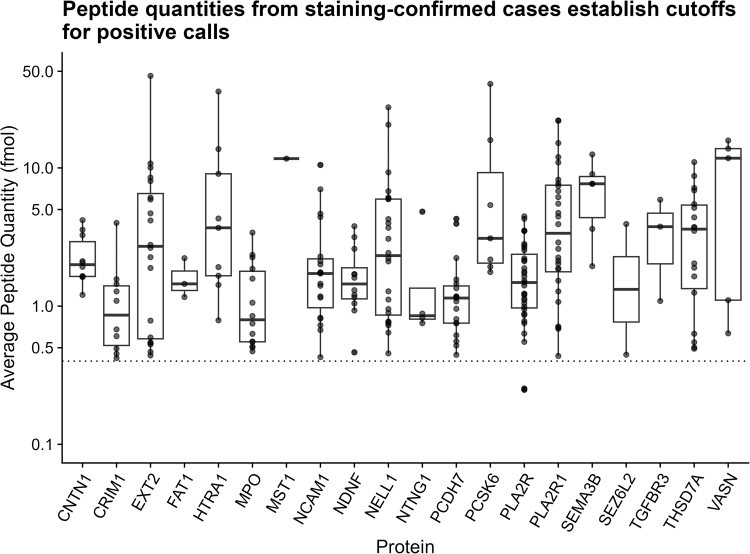
Comparison of fmol abundance assessed by standard peptides and accuracy of MS calls for various MN antigens. A cutoff of 0.4 fmol peptide was chosen as it provided high sensitivity while maintaining accuracy.

Antigen peptide detections were compared with the immunostaining results to empirically determine the cutoffs used in the calling algorithm that would result in optimal sensitivity and specificity (Figure 2). Similarly, IgG and IS recovery thresholds were determined empirically based on the accuracy of calls during preverification. From the analysis, a minimum IgG recovery was established as 100 ng (Figure 2a horizontal axis) and minimum IS intensity was 100,000 ion counts (Figure 2a vertical axis). The minimum peptide quantity of antigen through comparison of immunostaining-confirmed cases was 0.4 fmol (Figure 3). There were no false positives when 2 or more peptides at ≥ 0.4 fmol were detected for a single antigen. From this finding, we decided that when > 1 peptide is detected with adequate IgG capture and IS recovery, the results can be considered diagnostic and can be reported without the need for confirmatory immunohistochemical analysis.

Samples with IgG recovery below the 100 ng threshold were more likely to yield false negative results, but are unlikely to show false positivity for any antigen type (Figure 2b). In this defined region, samples with > 1 detected antigen peptide and sufficient IS signal but insufficient IgG recovery were still concordant with confirmation immunostaining. Therefore, test results with > 1 detected antigen peptide and sufficient IS recovery are called diagnostic with any quantity of IgG recovered. Test results with 0 antigen peptides detected and IgG recovery below the 100 ng threshold are called insufficient, and test results with 0 antigen peptides detected and IgG recovery above the 100 ng threshold are called negative.

In 2 scenarios, we perform immunostaining as a reflex test for confirmation of the target antigen. These include when a protein is detected without recovery of sufficient internal standards or when only 1 antigen peptide is detected. Cases where no peptides specific to any of the target proteins are detected and with adequate IgG capture and IS recovery are called negative (Table 1).

**Table 1:** Calling algorithm for membranous antigens

Peptides detected ≥ 0.4 fmol	IgG captured	IS recovered	IHC required	IHC result	Call
≥ 2	Any	≥ 100,000	No	N/A	Diagnostic
1	Any	≥ 100,000	Yes	Positive	Diagnostic
1	> 100 ng	≥ 100,000	Yes	Negative	Negative
1	< 100 ng	≥ 100,000	Yes	Negative	Insufficient
0	>100 ng	≥ 100,000	No	N/A	Negative
0	<100 ng	≥ 100,000	No	N/A	Insufficient
≥1	Any	< 100,000	Yes	Positive	Diagnostic
≥1	Any	< 100,000	Yes	Negative	Insufficient, repeat
0	Any	< 100,000	No	N/A	Insufficient, repeat

IS, internal standard; IHC, immunohistochemistry; MS, mass spectrometry; N/A, not applicable.

A calling algorithm for antigen typing was developed from the prevalidation studies. The defined rules maximize accuracy of the assay by establishing conditions where antigen type can be confidently called from the MS data, and additionally determining the criteria where immunostaining will confirm the presence or absence of an antigen.

### Clinical Validation and Assay Performance

After the development of a calling algorithm, a cohort of 110 MN cases of known antigen type (through use of immunostaining as the ‘ground truth’) and 8 cases of mesangial proliferative lupus nephritis were analyzed for clinical validation. The MN cases included those positive for PLA2R (*n* = 25), THSD7A (*n* = 9), EXT1/2 (*n* = 14), NELL1 (*n* = 11), NDNF (*n* = 6), PCDH7 (n = 10), NCAM1 (*n* = 9), myeloperoxidase (*n* = 4), semaphorin 3B (*n* = 3), CNTN1 (*n* = 5), HTRA1 (*n* = 3), CRIM1 (*n* = 4), netrin G1 (*n* = 1), vasorin (*n* = 2), macrophage-stimulating protein (*n* = 1), transforming growth factor beta receptor 3 (*n* = 1), protocadherin FAT1 (*n* = 1), and PCSK6 (*n* = 1). The cases of mesangial proliferative lupus nephritis were included as negative control samples, as they have glomerular IgG immune complex-deposits and are not cases of MN, and therefore, would be suspected to be negative for all antigens within the panel. Additionally, within each batch, a pooled diabetic glomerulosclerosis lysate was included as a negative control without IgG immune complex-deposits.

**Figure 4:**
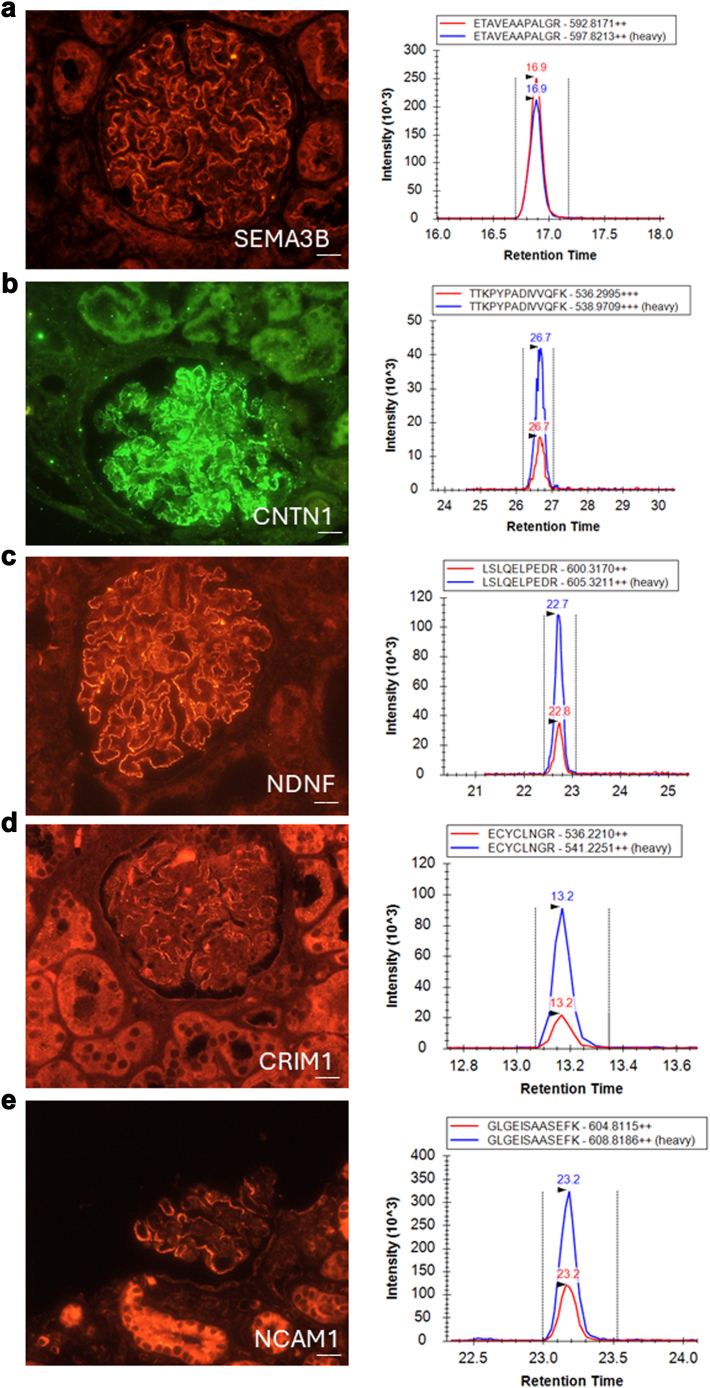
Representative endogenous peptide quantitation (red) by comparison to spiked standards (blue) within MN samples of unknown antigen type. All represent antigens of overall low frequency, and therefore, unlikely to be detected through immunostaining in standard panels as all comprise < 3% of total MN cases. (a–e) Detection of semaphorin 3B, CNTN1, NDNF, CRIM1, and NCAM1, respectively.

Cases were blinded following sample preparation and before the MS run. After MRM-MS, calls were made based upon the calling algorithm. Cases were unblinded and immunohistochemistry was performed for the identified target antigen(s) for confirmation of antigen type. After validation studies, we did not require immunostaining confirmation in every case as we did not have false positive MS results when there was ≥0.4 fmol antigen recovered with representation of ≥ 2 peptides. Representative cases are shown in Figure 4.

### Assay Performance

**Figure 5:**
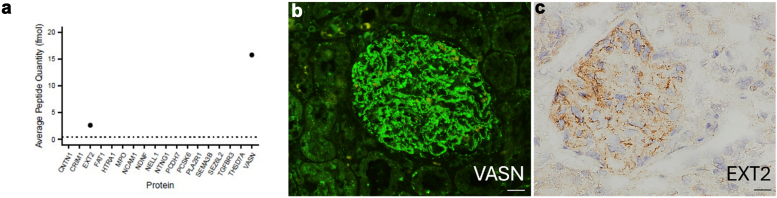
Representative dual positive case (EXT2+/VASN+). (a) Detection of endogenous analyte for VASN and EXT2 within the same sample. (b and c) Immunostaining confirmation for VASN and EXT2, respectively. VASN was of increased relative abundance as seen by a, typical for all dual-positive cases, which each had a dominant antigen and 1 of lower abundance. Scale bars = 20 μm. EXT, exostosin; VASN, vasorin.

Of 118 cases, 113 had sufficient IgG recovery (95.8%) and none had IS loss (IS < 100,000). Of the 107 adequate typed cases with sufficient IgG recovery, 104 were “diagnostic” for an antigen type (97.2%) and 3 were falsely called “negative” (2.8%) after applying the calling algorithm workflow. All cases “diagnostic” for antigen type had the corresponding antigen confirmed by immunostaining. Of the cases diagnosed as positive (including dual positives), 33 had 1 peptide detected and 70 had > 1 peptide. Antigens with 1 peptide call were often related to the number of validated IS peptides covering a given antigen, where in some cases we had included only 1 standard peptide. Extending the IS panel to improve coverage will minimize the need for confirmation staining. Four cases were positive for more than 1 antigen, including 1 case each positive for NELL1/CNTN1, PCDH7/PLA2R, EXT/vasorin, and EXT/PCSK6 and were confirmed by immunostaining results. A representative dual positive case is shown in Figure 5. The same cutoff of ≥ 0.4 fmol antigen recovery is required for each of the 2 antigens in dual positive cases.

The overall clinical performance in the validation cohort was as follows: accuracy of the workflow for assignment of the correct antigen type was 97.2%. The analytic sensitivity was 97.2%. The analytic specificity was 100%, as there were no false positive cases. Inter-run precision was assessed by running 12 aliquots of pooled PLA2R+ lysate on 10 separate days. The agreement between runs was 100%. The IS performance was also 100%.

### Diagnostic Yield in PLA2R-Negative MN

In an effort to determine the expected positivity rate in what we expect to be the primary clinical indication (biopsies with PLA2R-negative MN), we assessed a series of cases of consecutively diagnosed PLA2R-negative MN cases. Of 52 consecutive PLA2R negative samples, 49 had adequate IgG recovery (94.2%). Of these samples, a total of 28 (57.1%) were diagnosed as positive for an antigen. These included 10 NELL1, 6 THSD7A, 3 EXT1/2, 1 seizure-related 6 homolog 2, 2 CRIM1, 2 netrin G1, 2 HTRA1, 1 PCDH7, and 1 dual THSD7A/PCDH7 case. The remaining were negative for all antigens in the panel.

Of all biopsy-proven MN cases, PLA2R-positive cases make up approximately 55%.^19^ Taken together, incorporating this MN workflow would result in 81% of patients with MN having a diagnosed antigen type (given the detection rate of 57.1% of the expected 45% PLA2R-negative cases). Although not included in the validation studies, we have observed cases of PLA2R-negative MN by immunostaining identified to be positive by MS, which may demonstrate improved sensitivity over immunostaining rather than false positive results. This has been similarly reported by others.^20^^,^^21^

In parallel, we assessed 66 consecutive MLN cases, of which had a much lower frequency of autoantigen detection. Of these cases, 61 had adequate IgG and IS recovery (92.4%), with 11 having an identified antigen type (18.0%). These included 5 EXT1/2, 2 NCAM1, 1 CRIM1, 1 PCSK6, 1 transforming growth factor beta receptor 3, and 1 macrophage-stimulating protein case. The low yield of this assay in MLN may represent unknown autoantigens in lupus nephritis.

### Quality Control Procedures

We used matrix-matched quality control samples composed of defined quantities of each analyte to monitor MS performance drift. These included measuring 1, 5, and 20 fmol peptide standard controls. Analyte quality control samples enable system suitability checks and longitudinal performance monitoring. The quality control samples are used each day of testing to verify the analytical measuring range of each analyte and assess MS performance drift before running unknown samples, and stable longitudinal performance is observed (Supplementary Figure S1). Additionally, with each day of testing, we run an aliquot of a pooled PLA2R+ MN lysate and a control diabetic glomerulosclerosis lysate (PLA2R-) to assess pulldown reproducibility, which shows consistency among samples (Supplementary Figure S2). Results were consistent among 3 operators, with variation limited by use of semiautomation and detailed standard operating procedures.

## Discussion

**Figure 6:**
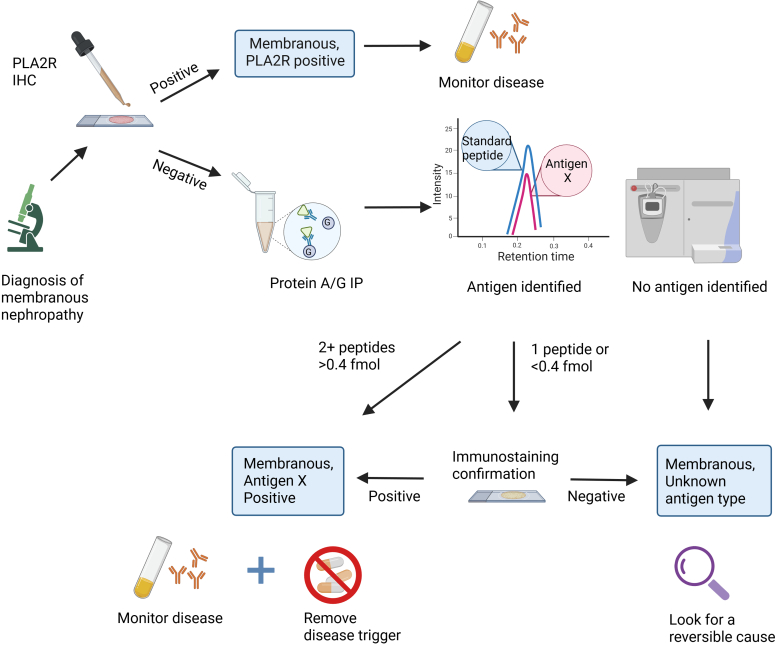
Workflow for deployment of the multiple reaction monitoring mass spectrometry (MRM-MS) MN assay into clinical practice. Following a diagnosis of PLA2R-negative MN, protein A/G IP is performed from residual frozen biopsy tissue, followed by interrogation of immune complexes by MRM-MS. MS results are subjected to the calling algorithm, with confidence in a positive call when two or more peptides are detected at > 0.4 fmol quantities. Below this threshold, a putative identification will be confirmed by immunostaining or may be negative for all antigens in the panel. IHC, immunohistochemistry; MN, membranous nephropathy; PLA2R, phospholipase A2 receptor.

MN classification has evolved over the past 20 years from a pattern of immunofluorescence staining diagnosed as either “idiopathic” or “secondary” based on disease associations to characterization at the molecular level based on the pathogenic antigen involved in disease. We now know at least 30 unique antigens involved in disease, and more are sure to follow. This precision-based understanding of disease presents both opportunities for better diagnosis as well as challenges associated with the limited tissue available in a needle core biopsy to perform the number of immunohistochemical assays for typing. We have developed an MRM-based MS workflow to address this problem through multiplexing detection of 19 total antigens with high accuracy, sensitivity, and specificity. We propose using this workflow on all MN cases that stain negative for PLA2R as it will enable a cost-effective method for precision diagnosis in patients with MN (Figure 6). However, up-front costs in bringing up this assay may be limiting to laboratories with low case volumes or where MS instrumentation is not available.

Determination of the antigenic target of autoantibodies in MN currently guides next steps in the diagnostic evaluation as a result of the known disease associations with many of the antigens. Examples include testing for syphilis (NDNF), assessing for nonsteroidal anti-inflammatory drugs use (PCSK6), evaluating for cancer or specific drug/heavy metal exposure (NELL1), to name a few. Additional associations with other rare antigens will likely be discovered in the future. Clinical utility studies of multiplex antigen typing assays are needed to determine the extent to which antigen typing impacts patient care. Ultimately, the hope for precision medicine is better patient outcomes by enabling more effective therapy. Testing antigen type in MN clinical trials will inform their significance for treatment strategies.

Use of a multiplexed panel will also allow for identification of dual antigen types which may be missed by standard immunostaining approaches. In a study of dual antigen MN cases identified by MS at the Mayo Clinic and at Arkana, approximately 3% of MN cases are of dual antigen type.^22^ MS has also been shown to have increased sensitivity compared with immunostaining, as reported by identifying cases of PLA2R-positive MN that are negative by immunostaining by two independent groups.^20^^,^^21^ We have also observed rare cases of PLA2R negative MN by immunostaining that were positive by MS, but were not present in our validation series.

Sethi *et al.* have also validated a multiplex MS-based workflow for diagnosis of MN antigens that currently detects a total of 13 antigens.^23^ There are several differences between their approach and those described here, although both methods can reliably identify an antigen type. The sample type used in our assay is the remaining unfixed tissue that is submitted for immunofluorescence whereas Sethi *et al.* use laser capture microdissected glomeruli from formalin-fixed paraffin embedded tissue. Each have unique advantages and disadvantages and are complementary approaches. Using unfixed tissue enables processing by immunoprecipitation and thus avoiding the laborious laser capture microdissection procedure, but will capture extraglomerular immune complexes rather than those restricted to glomeruli. However, we do not anticipate that capture of extraglomerular immune complexes (along tubular basement membranes or vessels) would be a significant problem, as within our validation studies, as well as an additional 420 cases reported previously, there was complete concordance with immunostaining results.^3^ The use of immunoprecipitation reduces background proteins compared with laser capture microdissected, which detects all proteins within glomeruli. This is often not a significant barrier, but podocyte proteins would be detected at low levels when analyzing all glomerular proteins with laser capture microdissected (PLA2R, THSD7A, HTRA1, etc.). Another difference is the MS approach. The assay described here is an MRM approach that uses a triple quadripole MS platform with IS peptides whereas Sethi *et al.* use an oribitrap mass spectrometer without internal standards published as part of their workflow, with peptide-spectrum-match counting used in measurements. The diagnostic yield was similar between the two assays 57.1% in our assay compared with 49.1% from the Mayo assay.^23^ It is suspected that both panels will improve over new iterations as additional antigens are incorporated.

This assay will enable future research to further characterize MN. Using our workflow, approximately 19% of MN cases remain uncharacterized. Therefore, we can now enrich our renal biopsies for future discovery work to identify additional antigenic drivers of disease. There are currently subsets of MN with a significant knowledge gap remaining of antigen types, including malignancy-associated MN, MLN, and MN in the pediatric population. As MS becomes integrated into clinical practice, cohorts of patients with rare or new antigens that are identified will allow for further characterization of each disease in the future.

### Limitations

Although the assay multiplexes 19 different antigens, there are more than 10 antigens that are unable to be detected using this assay. In its current state, it is an improvement over existing immunohistochemical typing methods, however it can be further improved to require less starting material and to cover more antigens. The assay would not guard against *in vitro* formation of antigen-antibody complexes. However, in healthy conditions, autoantibodies should not be produced directed against podocyte or other MN antigens, and therefore, we do not anticipate that this will create false positive results. An external validation study has not been performed; however, this assay is complex to establish in a new laboratory and the methodology should be sufficiently detailed to facilitate adoption.

### Future Directions

A clinical utility study is underway to examine the impact of a more comprehensive approach to antigen typing on clinical outcomes, diagnostic impact, and cost-effectiveness. The clinical utility of antigen typing in MN is well-established for antigens with known disease associations, such as NDNF with syphilis, PCSK6 with nonsteroidal anti-inflammatory drugs use, NELL1 with malignancy and various medications, and EXT1/2 with MLN. However, this needs to be evaluated in a greater context to quantify the overall impact following incorporation into clinical practice compared with the previous standard of care of a limited panel of immunostains used in typing cases.

In summary, we describe here a novel MS workflow to multiplex the diagnosis of 19 different MN antigen types using MRM-based MS on immunopreciptates from frozen kidney biopsy lysates. The use of immunopreciptation to capture immune complexes enables a sample processing workflow that scales with larger batch sizes. The MRM approach with internal standards and empirically determined quantitative cutoffs for making antigen diagnosis calls allows for accurate and reproducible results that comply with the rigorous quality assurance and quality control requirements of a chemiluminescence immunoassay laboratory. The antigenic classification will enable a new era of diagnosis, monitoring, and treatment of MN for patients.

## Disclosure

All the authors declared no competing interests.

## Supplementary Material

Supplementary file (PDF) **Supplementary Methods. Figure S1.** Quality control samples of standard peptide mixtures run at 1, 5, and 20 fmol are used for system suitability checks and demonstrate stable longitudinal performance. **Figure S2.** Protein A/G immunoprecipitation efficiency assessed through evaluating a pooled lysate of PLA2R+ MN samples. **Table S1.** Limits of detection for all internal standard peptides. **Table S2.** Interassay variation, intraassay variation, and evaluation of matrix effects of all standard peptides. **Table S3.** Liquid chromatography gradient parameters.

## Footnotes

Supplementary file (PDF)

**Supplementary Methods.**
**Figure S1.** Quality control samples of standard peptide mixtures run at 1, 5, and 20 fmol are used for system suitability checks and demonstrate stable longitudinal performance. **Figure S2.** Protein A/G immunoprecipitation efficiency assessed through evaluating a pooled lysate of PLA2R+ MN samples. **Table S1.** Limits of detection for all internal standard peptides. **Table S2.** Interassay variation, intraassay variation, and evaluation of matrix effects of all standard peptides. **Table S3.** Liquid chromatography gradient parameters.
