# Supplementary material for: Multiplexed Detection of Membranous Nephropathy Antigens by Multi-Reaction Monitoring Mass Spectrometry
Source: Kidney Int Rep. 2026 Apr 22;11(7):106561. doi: 10.1016/j.ekir.2026.106561 (PMC13235345; doi:10.1016/j.ekir.2026.106561)
Supplement: Supplementary file (PDF) — Supplementary Methods. Figure S1. Quality control samples of standard peptide mixtures run at 1, 5, and 20 fmol are used for system suitability checks and demonstrate stable longitudinal performance. Figure S2. Protein A/G immunoprecipitation efficiency assessed through evaluating a pooled lysate of PLA2R+ MN samples. Table S1. Limits of detection for all internal standard peptides. Table S2. Interassay variation, intraassay variation, and evaluation of matrix effects of all standard peptides. Table S3. Liquid chromatography gradient parameters. [file mmc1.pdf]

Data Supplement:

Table of Contents.

Supplementary Methods..1

Supplementary Figures..2-3

Supplementary Figure S1. Quality control samples of standard peptide mixtures run at 1, 5, and 20 fmol are used for system suitability checks and demonstrate stable longitudinal performance.

Supplementary Figure S2. Protein A/G immunoprecipitation reproducibility assessed through evaluating a pooled lysate of PLA2R+ MN samples.

Supplementary Tables..3-8

Supplementary Table S1. Limits of detection for all internal standard peptides

Supplementary Table S2. Interassay variation, intraassay variation, and evaluation of matrix effects of all standard peptides.

Supplementary Table S3. Liquid chromatography gradient parameters

Supplementary Methods.

Recombinant proteins and overexpression cell lysates for determination of internal standard peptides. Protein sources were as follows: PLA2R (LS Bio), THSD7A (R+D systems), EXT2 (R+D systems), NELL1 (R+D systems), PCSK6 (R+D systems), NTNG1 (Sinobiological), MPO (R + D systems), SEMA3B (R+D systems), PCDH7 (Novus Biologicals), TGFBR3 (R + D systems), NCAM1 (R + D systems), SEZ6L2 (AbCam), MST1 (R + D systems), VASN (R + D systems), NDNF (Abbexa), HTRA1 (R + D systems), CRIM1 (R + D), and FAT1 (Novus Biologicals).

Overexpression HEK293T cell lysates were also examined, but not available for all antigenic targets. These included SEMA3B, PCDH7, HTRA1, TGFBR3, NCAM1, CNTN1, SEZ6L2, MST1, VASN, NTNG1, PCSK6, NDNF, MPO (OriGene).

Performance characteristics for each chosen peptide were evaluated by the Clinical Proteomic Tumor Analysis Consortium Guidelines. Limits of detection are shown in Supplementary Table S1. Intra- and interassay CVs and evaluation of matrix effects are included in Supplementary Table S2.

Antibodies and staining conditions. Antibodies used in immunostaining to confirm antigen types are included in the table below. We utilized tissues with endogenous expression for each protein (pancreas, liver, skin, colon, etc) as positive controls.

Antibody	Source	Species	Dilution	Endogenous control

PLA2R1	Sigma, cat # HPA012657	Rabbit polyclonal	1:25	Podocytes
THSD7A	Thermo-Fisher, cat # PA5-51486	Rabbit polyclonal	1:200	Podocytes
NELL1	Novus Biologicals, cat # H00004745-M01	Mouse monoclonal	1:200	Liver
EXT1	AbCam, cat # ab126305	Rabbit polyclonal	1:50	Liver, colon
EXT2	AbCam, cat # ab203843	Rabbit polyclonal	1:50	Liver, colon
NCAM1	Sigma, cat # HPA039835	Rabbit polyclonal	1:50	Brain
CNTN1	R + D Systems, cat # AF904	Goat polyclonal	1:50	Pituitary
NDNF	LSBio, cat # LS-C168140	Rabbit polyclonal	1:100	Eye (retina)
MPO	Sigma, cat # 475915	Rabbit polyclonal	1:200	Pyelonephritis
SEZ6L2	Thermo-Fisher, cat # PA5-64172	Rabbit polyclonal	1:50	Skin
NTNG1	Santa Cruz, cat # sc-271774	Mouse monoclonal	1:100	Skin
CRIM1	Thermo Fisher, cat # PA5-51412	Rabbit polyclonal	1:50	Pancreas
PCDH7	Thermo Fisher, cat # PA5-52862	Rabbit polyclonal	1:200	Colon, tonsil
HTRA1	Bio-techne, cat # MAB29161	Mouse monoclonal	1:50	Colon, pancreas
MST1	Thermo-Fisher, cat # PA5-42762	Rabbit polyclonal	1:50	Liver, pancreas
VASN	Bio-techne, cat # AF2140	Goat polyclonal	1:50	Pancreas (islets)
SEMA3B	AbCam, cat # ab48197	Rabbit polyclonal	1:800	Skin, Testis
TGFBR3	Sigma, cat # HPA008257	Rabbit polyclonal	1:25	Pancreas
PCSK6	Thermo Fisher, cat # PA5-32966	Rabbit polyclonal	1:50	Pancreas
FAT1	AbCam, cat # ab98892	Rabbit polyclonal	1:200	Skin, Colon

Supplementary Figures.

Supplementary Figure S1. Quality control samples of standard peptide mixtures run at 1, 5, and 20 fmol are used for system suitability checks and demonstrate stable longitudinal performance.

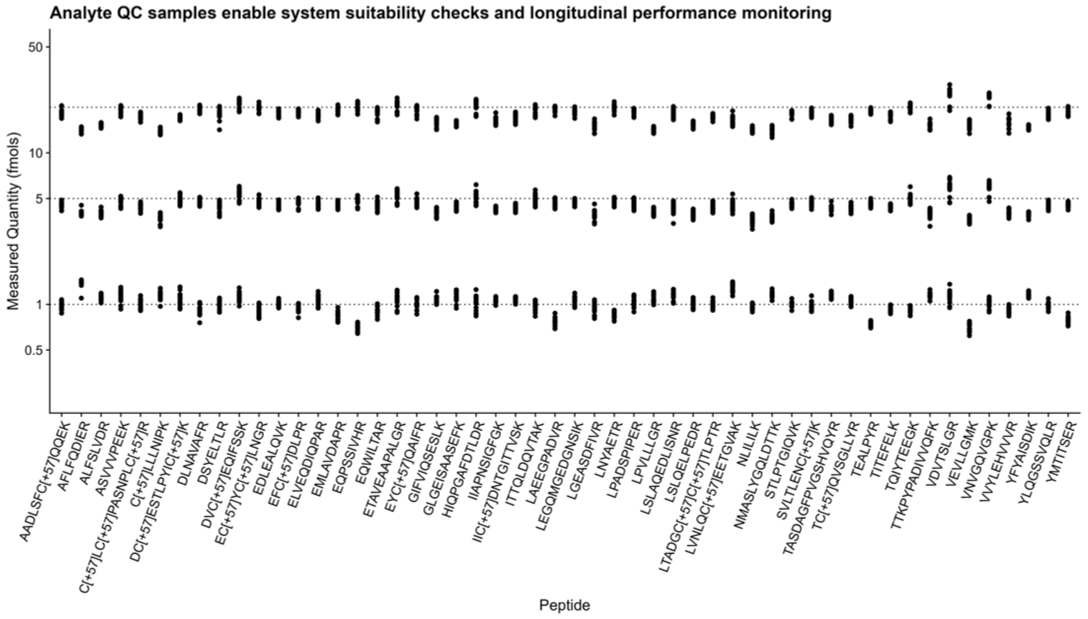

Supplementary Figure S2. Protein A/G immunoprecipitation reproducibility assessed through evaluating a pooled lysate of PLA2R+ MN samples. Daily measurements over eight weeks are shown, demonstrating consistency across measurements.

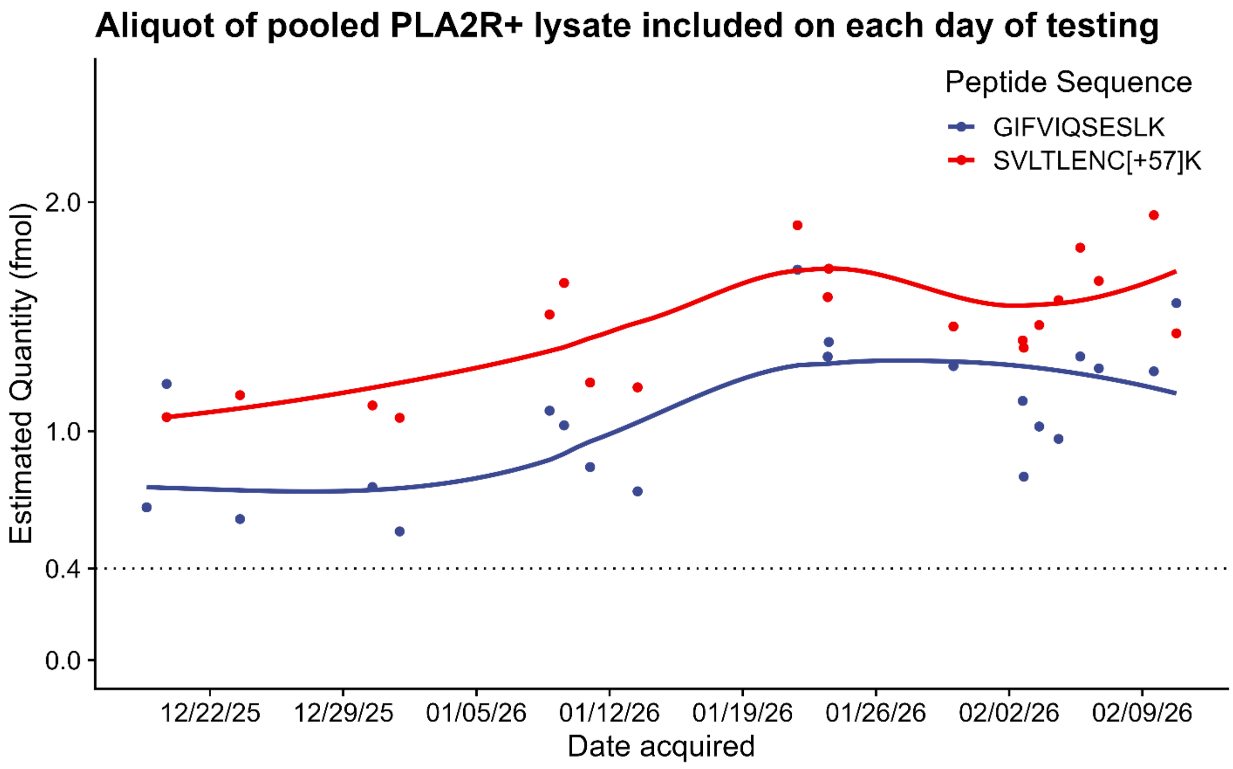

Supplementary Tables.

Supplementary Table S1. Limits of detection for all internal standard peptides

Protein	Peptide	Limit of Detection	R squared
CNTN1	TTKPYPADIVVQFK	0.254	0.999
CRIM1	LTADGCCTLPTR	0.333	0.997
CRIM1	ECYCLNGR	0.27	0.998
EXT2	LPADSPIPER	0.276	0.998
EXT2	EDLEALQVK	0.334	0.998
EXT2	ASVVVPEEK	0.322	0.998
FAT1	LVNLQCEETGVAK	0.211	0.994
FAT1	STLPTGIQVK	0.27	0.998
HTRA1	YNFIADVVEK	0.267	0.999
HTRA1	LPVLLLGR	0.216	1
MST1	EFCDLPR	0.243	0.998
MST1	EQWILTAR	0.404	0.997
NCAM1	GLGEISAASEFK	0.254	0.999
NCAM1	LEGQMGEDGNSIK	0.315	0.996
NDNF	LSLQELPEDR	0.257	0.999

NDNF	VDVTSLGR	0.387	0.998
NELL1	AFLFQDIER	0.263	0.999
NELL1	TEALPYR	0.266	0.999
NELL1	LNYAETR	0.228	0.999
NELL1	TCQVSGLLYR	0.242	0.999
NTNG1	TQIYTEEGK	0.214	0.998
NTNG1	NMASLYGQLDTTK	0.252	0.998
NTNG1	YFYAISDIK	0.228	0.999
PCDH7	LAEEGPADVR	0.271	0.998
PCDH7	DSYELTLR	0.316	0.996
PCSK6	VVYLEHVVVR	0.232	0.999
PLA2R	GIFVIQSESLK	0.28	0.998
PLA2R	SVLTLENCK	0.281	0.999
PLA2R	DCESTLPYICK	0.265	0.998
PLA2R	CLLLNIPK	0.226	0.998
SEMA3B	ETAVEAAPALGR	0.278	0.999
SEZ6L2	TASDAGFPVGSHVQYR	0.321	0.996
TGFBR3	NLILILK	0.184	0.999
TGFBR3	IIAPNSIGFGK	0.296	0.998
THSD7A	AADLSFCQQEK	0.264	0.999
THSD7A	SILAYAGEEGGIR	0.199	0.993
THSD7A	VNVGQVGPK	0.254	0.999
THSD7A	VEVLLGMK	0.249	0.998
VASN	YLQGSSVQLR	0.302	0.999

Supplementary Table S2. Interassay variation, intraassay variation, and evaluation of matrix effects of all standard peptides.

Protein	Peptide	Amount	Interassay CV	Intraassay CV	Total CV	Intramatrix CV	Intermatrix CV	Total CV
CNTN1	ELTITWAPLSR	1fmol	0.071	0.066	0.097	0.045	0.098	0.108
CNTN1	ELTITWAPLSR	5fmol	0.056	0.073	0.092	0.018	0.06	0.063
CNTN1	ELTITWAPLSR	25fmol	0.019	0.019	0.027	0.016	0.076	0.078
CNTN1	TTKPYPADIVVQFK	1fmol	0.033	0.023	0.04	0.029	0.05	0.058
CNTN1	TTKPYPADIVVQFK	5fmol	0.011	0.02	0.023	0.018	0.047	0.05
CNTN1	TTKPYPADIVVQFK	25fmol	0.009	0.018	0.021	0.011	0.03	0.032
CRIM1	ECYCLNGR	1fmol	0.019	0.02	0.027	0.022	0.022	0.031
CRIM1	ECYCLNGR	5fmol	0.015	0.008	0.018	0.013	0.021	0.024
CRIM1	ECYCLNGR	25fmol	0.013	0.012	0.018	0.02	0.02	0.028
CRIM1	LTADGCCTLPTR	1fmol	0.034	0.025	0.042	0.032	0.039	0.05
CRIM1	LTADGCCTLPTR	5fmol	0.008	0.009	0.012	0.023	0.023	0.032
CRIM1	LTADGCCTLPTR	25fmol	0.01	0.019	0.021	0.012	0.023	0.026
CRIM1	VLCETEVCPPLLCQNPSR	1fmol	0.084	0.084	0.119	0.087	0.115	0.145
CRIM1	VLCETEVCPPLLCQNPSR	5fmol	0.047	0.046	0.066	0.03	0.055	0.063

CRIM1	VLCETEVCPPLLCQNPSR	25fmol	0.037	0.038	0.052	0.023	0.061	0.065
EXT2	ASVVVPEEK	1fmol	0.02	0.027	0.033	0.021	0.028	0.035
EXT2	ASVVVPEEK	5fmol	0.01	0.012	0.015	0.023	0.024	0.033
EXT2	ASVVVPEEK	25fmol	0.013	0.015	0.02	0.023	0.022	0.032
EXT2	EDLEALQVK	1fmol	0.032	0.038	0.049	0.019	0.032	0.037
EXT2	EDLEALQVK	5fmol	0.014	0.013	0.019	0.014	0.024	0.028
EXT2	EDLEALQVK	25fmol	0.01	0.01	0.014	0.012	0.014	0.018
EXT2	FASVFGTMPLK	1fmol	0.079	0.062	0.1	0.044	0.097	0.106
EXT2	FASVFGTMPLK	5fmol	0.025	0.027	0.037	0.021	0.061	0.065
EXT2	FASVFGTMPLK	25fmol	0.024	0.022	0.032	0.022	0.052	0.057
EXT2	LPADSPIPER	1fmol	0.018	0.015	0.023	0.025	0.031	0.04
EXT2	LPADSPIPER	5fmol	0.018	0.018	0.025	0.018	0.025	0.031
EXT2	LPADSPIPER	25fmol	0.004	0.016	0.017	0.015	0.015	0.021
FAT1	LVNLQCEETGVAK	1fmol	0.035	0.04	0.053	0.047	0.057	0.074
FAT1	LVNLQCEETGVAK	5fmol	0.033	0.037	0.049	0.032	0.043	0.053
FAT1	LVNLQCEETGVAK	25fmol	0.02	0.025	0.032	0.03	0.049	0.057
FAT1	STLPTGIQVK	1fmol	0.021	0.021	0.029	0.018	0.033	0.038
FAT1	STLPTGIQVK	5fmol	0.018	0.018	0.025	0.017	0.022	0.028
FAT1	STLPTGIQVK	25fmol	0.011	0.015	0.019	0.012	0.017	0.021
MST1	EFCDLPR	1fmol	0.032	0.036	0.048	0.035	0.036	0.05
MST1	EFCDLPR	5fmol	0.021	0.025	0.033	0.01	0.018	0.021
MST1	EFCDLPR	25fmol	0.018	0.021	0.027	0.015	0.019	0.024
MST1	EQWILTAR	1fmol	0.029	0.036	0.046	0.027	0.049	0.055
MST1	EQWILTAR	5fmol	0.011	0.017	0.02	0.01	0.023	0.025
MST1	EQWILTAR	25fmol	0.011	0.011	0.016	0.01	0.016	0.019
HTRA1	LPVLLLGR	1fmol	0.027	0.03	0.041	0.018	0.045	0.049
HTRA1	LPVLLLGR	5fmol	0.009	0.016	0.019	0.011	0.026	0.028
HTRA1	LPVLLLGR	25fmol	0.012	0.019	0.022	0.012	0.028	0.03
HTRA1	YNFIADVVEK	1fmol	0.042	0.044	0.061	0.019	0.058	0.061
HTRA1	YNFIADVVEK	5fmol	0.025	0.038	0.045	0.019	0.039	0.043
HTRA1	YNFIADVVEK	25fmol	0.022	0.026	0.034	0.01	0.039	0.04
NCAM1	FIVLSNNYLQIR	1fmol	0.172	0.384	0.421	0.091	0.152	0.177
NCAM1	FIVLSNNYLQIR	5fmol	0.07	0.068	0.098	0.056	0.087	0.103
NCAM1	FIVLSNNYLQIR	25fmol	0.077	0.095	0.123	0.06	0.11	0.125
NCAM1	GLGEISAASEFK	1fmol	0.023	0.031	0.039	0.02	0.034	0.04
NCAM1	GLGEISAASEFK	5fmol	0.015	0.014	0.02	0.008	0.028	0.029
NCAM1	GLGEISAASEFK	25fmol	0.009	0.015	0.018	0.01	0.032	0.034
NCAM1	LEGQMGEDGNSIK	1fmol	0.046	0.041	0.061	0.041	0.055	0.069
NCAM1	LEGQMGEDGNSIK	5fmol	0.031	0.035	0.047	0.016	0.021	0.027
NCAM1	LEGQMGEDGNSIK	25fmol	0.015	0.028	0.032	0.013	0.013	0.018
NDNF	LSLQELPEDR	1fmol	0.035	0.055	0.065	0.021	0.056	0.06
NDNF	LSLQELPEDR	5fmol	0.016	0.014	0.021	0.01	0.035	0.036
NDNF	LSLQELPEDR	25fmol	0.012	0.01	0.016	0.008	0.035	0.036
NDNF	VDVTSLGR	1fmol	0.039	0.023	0.045	0.03	0.056	0.064

NDNF	VDVTSLGR	5fmol	0.03	0.032	0.044	0.024	0.032	0.04
NDNF	VDVTSLGR	25fmol	0.015	0.017	0.022	0.016	0.021	0.027
NELL1	AFLFQDIER	1fmol	0.021	0.028	0.035	0.021	0.062	0.066
NELL1	AFLFQDIER	5fmol	0.021	0.022	0.031	0.012	0.041	0.043
NELL1	AFLFQDIER	25fmol	0.011	0.016	0.02	0.013	0.055	0.056
NELL1	LNYAETR	1fmol	0.028	0.029	0.041	0.021	0.03	0.037
NELL1	LNYAETR	5fmol	0.012	0.015	0.019	0.016	0.023	0.028
NELL1	LNYAETR	25fmol	0.01	0.01	0.014	0.014	0.02	0.025
NELL1	TCQVSGLLYR	1fmol	0.02	0.024	0.032	0.014	0.034	0.037
NELL1	TCQVSGLLYR	5fmol	0.013	0.013	0.018	0.009	0.023	0.025
NELL1	TCQVSGLLYR	25fmol	0.009	0.012	0.015	0.01	0.016	0.019
NELL1	TEALPYR	1fmol	0.011	0.014	0.018	0.016	0.025	0.029
NELL1	TEALPYR	5fmol	0.01	0.015	0.018	0.008	0.012	0.014
NELL1	TEALPYR	25fmol	0.005	0.014	0.015	0.013	0.018	0.022
NTNG1	FAFFAGPR	1fmol	0.036	0.021	0.042	0.016	0.043	0.046
NTNG1	FAFFAGPR	5fmol	0.023	0.02	0.031	0.012	0.034	0.036
NTNG1	FAFFAGPR	25fmol	0.023	0.022	0.032	0.011	0.018	0.021
NTNG1	NMASLYGGQLDTTK	1fmol	0.023	0.029	0.037	0.028	0.055	0.062
NTNG1	NMASLYGQLDTTK	5fmol	0.03	0.031	0.043	0.015	0.043	0.046
NTNG1	NMASLYGQLDTTK	25fmol	0.016	0.022	0.027	0.017	0.041	0.044
NTNG1	TQIYTEEGK	1fmol	0.029	0.031	0.043	0.026	0.029	0.038
NTNG1	TQIYTEEGK	5fmol	0.014	0.02	0.025	0.025	0.028	0.037
NTNG1	TQIYTEEGK	25fmol	0.015	0.014	0.02	0.016	0.019	0.025
NTNG1	YFYAISDIK	1fmol	0.024	0.028	0.037	0.011	0.053	0.054
NTNG1	YFYAISDIK	5fmol	0.017	0.024	0.03	0.01	0.035	0.037
NTNG1	YFYAISDIK	25fmol	0.01	0.011	0.015	0.01	0.037	0.038
PCDH7	DSYELTLR	1fmol	0.028	0.041	0.049	0.018	0.02	0.027
PCDH7	DSYELTLR	5fmol	0.01	0.01	0.014	0.006	0.01	0.011
PCDH7	DSYELTLR	25fmol	0.007	0.007	0.01	0.008	0.01	0.013
PCDH7	LAEEGPADVR	1fmol	0.033	0.034	0.047	0.022	0.028	0.035
PCDH7	LAEEGPADVR	5fmol	0.014	0.015	0.02	0.016	0.026	0.031
PCDH7	LAEEGPADVR	25fmol	0.018	0.018	0.025	0.016	0.021	0.026
PCDH7	VATVLATDADSGK	1fmol	0.028	0.04	0.049	0.026	0.036	0.045
PCDH7	VATVLATDADSGK	5fmol	0.013	0.015	0.02	0.016	0.025	0.029
PCDH7	VATVLATDADSGK	25fmol	0.017	0.02	0.026	0.018	0.019	0.026
PCDH7	VLITDVNDNSPR	1fmol	0.053	0.067	0.085	0.041	0.045	0.061
PCDH7	VLITDVNDNSPR	5fmol	0.047	0.053	0.071	0.066	0.072	0.097
PCDH7	VLITDVNDNSPR	25fmol	0.074	0.09	0.117	0.06	0.081	0.101
PCSK6	NVVVTILDDGIER	1fmol	0.106	0.106	0.15	0.079	0.115	0.139
PCSK6	NVVVTILDDGIER	5fmol	0.057	0.054	0.079	0.049	0.103	0.114
PCSK6	NVVVTILDDGIER	25fmol	0.047	0.058	0.075	0.046	0.104	0.114
PCSK6	VVYLEHVVVR	1fmol	0.032	0.033	0.046	0.032	0.04	0.051
PCSK6	VVYLEHVVVR	5fmol	0.012	0.018	0.022	0.015	0.029	0.033
PCSK6	VVYLEHVVVR	25fmol	0.011	0.012	0.017	0.013	0.015	0.02

PLA2R	CLLLNIPK	1fmol	0.036	0.03	0.047	0.026	0.083	0.087
PLA2R	CLLLNIPK	5fmol	0.026	0.035	0.044	0.014	0.04	0.042
PLA2R	CLLLNIPK	25fmol	0.016	0.02	0.026	0.021	0.035	0.041
PLA2R	DCESTLPYICK	1fmol	0.028	0.026	0.038	0.026	0.058	0.064
PLA2R	DCESTLPYICK	5fmol	0.018	0.021	0.027	0.015	0.035	0.038
PLA2R	DCESTLPYICK	25fmol	0.02	0.026	0.033	0.008	0.033	0.034
PLA2R	GIFVIQSESLK	1fmol	0.036	0.055	0.066	0.044	0.039	0.058
PLA2R	GIFVIQSESLK	5fmol	0.011	0.016	0.02	0.032	0.043	0.054
PLA2R	GIFVIQSESLK	25fmol	0.02	0.024	0.031	0.019	0.043	0.047
PLA2R	SVLTLENCK	1fmol	0.018	0.019	0.026	0.02	0.033	0.038
PLA2R	SVLTLENCK	5fmol	0.014	0.014	0.019	0.019	0.023	0.029
PLA2R	SVLTLENCK	25fmol	0.016	0.017	0.023	0.013	0.017	0.021
SEZ6L2	EGDMLTLFDGDGPSAR	1fmol	0.154	0.161	0.223	0.049	0.113	0.123
SEZ6L2	EGDMLTLFDGDGPSAR	5fmol	0.048	0.049	0.069	0.032	0.076	0.082
SEZ6L2	EGDMLTLFDGDGPSAR	25fmol	0.041	0.037	0.055	0.019	0.06	0.062
SEZ6L2	TASDAGFPVGSHVQYR	1fmol	0.036	0.033	0.049	0.064	0.064	0.09
SEZ6L2	TASDAGFPVGSHVQYR	5fmol	0.028	0.018	0.033	0.027	0.04	0.048
SEZ6L2	TASDAGFPVGSHVQYR	25fmol	0.018	0.022	0.028	0.024	0.025	0.035
SEMA3B	DFPDDVIQFAR	1fmol	0.086	0.102	0.133	0.06	0.088	0.107
SEMA3B	DFPDDVIQFAR	5fmol	0.029	0.034	0.045	0.036	0.089	0.095
SEMA3B	DFPDDVIQFAR	25fmol	0.016	0.025	0.03	0.029	0.097	0.101
SEMA3B	ETAVEAAPALGR	1fmol	0.022	0.025	0.033	0.018	0.022	0.028
SEMA3B	ETAVEAAPALGR	5fmol	0.01	0.018	0.02	0.011	0.016	0.019
SEMA3B	ETAVEAAPALGR	25fmol	0.01	0.015	0.018	0.015	0.016	0.022
TGFBR3	EYGAVTSFTELK	1fmol	0.017	0.016	0.023	0.01	0.059	0.06
TGFBR3	EYGAVTSFTELK	5fmol	0.019	0.026	0.032	0.009	0.039	0.04
TGFBR3	EYGAVTSFTELK	25fmol	0.014	0.015	0.021	0.01	0.042	0.044
TGFBR3	IIAPNSIGFGK	1fmol	0.017	0.016	0.023	0.025	0.034	0.043
TGFBR3	IIAPNSIGFGK	5fmol	0.02	0.023	0.031	0.038	0.055	0.067
TGFBR3	IIAPNSIGFGK	25fmol	0.015	0.018	0.024	0.038	0.039	0.054
TGFBR3	NLILILK	1fmol	0.029	0.023	0.037	0.031	0.031	0.044
TGFBR3	NLILILK	5fmol	0.015	0.015	0.021	0.012	0.041	0.043
TGFBR3	NLILILK	25fmol	0.02	0.022	0.03	0.016	0.028	0.032
THSD7A	AADLSFCQQEK	1fmol	0.026	0.027	0.038	0.028	0.047	0.054
THSD7A	AADLSFCQQEK	5fmol	0.019	0.022	0.029	0.026	0.031	0.041
THSD7A	AADLSFCQQEK	25fmol	0.025	0.025	0.035	0.019	0.021	0.028
THSD7A	SILAYAGEEGGIR	1fmol	0.026	0.034	0.043	0.026	0.078	0.082
THSD7A	SILAYAGEEGGIR	5fmol	0.013	0.021	0.025	0.014	0.051	0.053
THSD7A	SILAYAGEEGGIR	25fmol	0.015	0.015	0.021	0.008	0.044	0.045
THSD7A	VEVLLGMK	1fmol	0.038	0.041	0.056	0.016	0.018	0.023
THSD7A	VEVLLGMK	5fmol	0.018	0.019	0.026	0.012	0.019	0.023
THSD7A	VEVLLGMK	25fmol	0.018	0.017	0.025	0.009	0.012	0.015
THSD7A	VNVGQVGPK	1fmol	0.02	0.02	0.028	0.021	0.032	0.038
THSD7A	VNVGQVGPK	5fmol	0.023	0.024	0.033	0.025	0.035	0.043

THSD7A	VNVGQVGPK	25fmol	0.011	0.012	0.016	0.035	0.033	0.049
VASN	HIQPGAFDTLDR	1fmol	0.034	0.038	0.05	0.052	0.082	0.097
VASN	HIQPGAFDTLDR	5fmol	0.018	0.02	0.027	0.032	0.053	0.063
VASN	HIQPGAFDTLDR	25fmol	0.011	0.018	0.021	0.025	0.039	0.046
VASN	YLQGSSVQLR	1fmol	0.025	0.024	0.035	0.032	0.037	0.049
VASN	YLQGSSVQLR	5fmol	0.017	0.018	0.024	0.017	0.027	0.031
VASN	YLQGSSVQLR	25fmol	0.014	0.012	0.018	0.02	0.021	0.028

Supplementary Table S3. Liquid chromatography gradient parameters

Time	Flow (µL/min)	%A	%B
Initial	1.2	99.5	0.5
5.75	1.2	99.5	0.5
6	0.4	99.5	0.5
7.5	0.4	93.5	6.5
25	0.4	84	16
33.5	0.4	62	38
34	0.4	5	95
34.5	1.2	5	95
36	1.2	5	95
36.1	1.2	99.5	0.5

*Buffer A is comprised of 0.1% formic acid and 0.5% acetonitrile in Optima™ LC-MS Grade water. Buffer B consists of 0.1% formic acid in acetonitrile. The loop offline is 5.5 minutes.
